# The Difference between Aesthetic Appreciation of Artistic and Popular Music: Evidence from an fMRI Study

**DOI:** 10.1371/journal.pone.0165377

**Published:** 2016-11-04

**Authors:** Ping Huang, Hanhua Huang, Qiuling Luo, Lei Mo

**Affiliations:** 1 Center for Studies of Psychological Application, South China Normal University, Guangzhou, China; 2 School of Music, South China Normal University, Guangzhou, China; National University of Singapore, SINGAPORE

## Abstract

To test the hypothesis that pleasure from artistic music is intellectual while that from popular music is physiological, this study investigated the different functional mechanisms between aesthetic appreciation of artistic and popular music using fMRI. 18 male non-musicians were scanned while they performed an aesthetic rating task for excerpts of artistic music, popular music and musical notes playing and singing (control). The rating scores of artistic and popular music excerpts were both significantly higher than that of control materials while the scores of them were not different. The fMRI results showed both artistic and popular conditions activated the VS and vmPFC, compared with control condition. When contrasted popular and artistic condition directly, we found popular music activated right putamen, while artistic music activated right mPFC. By parametric analysis, we found the activation of right putamen tracked the aesthetic ratings of popular music, whereas the BOLD signal in right mPFC tracked the aesthetic ratings of artistic music. These results indicate the reward induced by popular music is closer to a primary reward while that induced by artistic music is closer to a secondary reward. We also found artistic music activated ToM areas, including PCC/PC, arMFC and TPJ, when compared with popular music. And these areas also tracked aesthetic ratings of artistic music but not those of popular music. These results imply that the pleasure from former comes from cognitive empathy. In conclusion, this study gives clear neuronal evidences supporting the view that artistic music is of intelligence and social cognition involved while the popular music is of physiology.

## Introduction

The aesthetics of artistic work has long been considered as disinterested, detached and intellectual, while that of popular work is perceptual and physical [1–3]. However, such speculative perspective is far from the essential characteristic within diverse levels of aethetics. Due to the advancement of neuroscience technologies, more researchers were committed to explore the neural basic of aesthetics, emerging a new research field of “Neuroaesthetics”. In the early period of the 1900s, the neural mechanism of aesthetic judgment or preference for artistic objects attracted the more interest of neuroscientists[4]. It was found that brain regions recruited by artistic appreciation were highly overlapped with the reward circuit. For example, OFC and ventral striatum which are two typical reward regions were found to be activated during the appreciation of beautiful paintings[5–7]. Recently, researchers have tried to figure out the universal brain correlates for more extensive aesthetics. In research by Ishizu and Zeki, the arousals of mOFC and ventral striatum was reported for both the visual (artistic painting) and acoustic (music) aesthetics[6]. Furthermore, Brown and his colleagues applied the meta analyses of activation likelihood estimation to demonstrate a core circuit for positive-valence aesthetic appraisal[8]. The anterior insular, NAcc, pregenual ACC, anterior midcingulate cortex, dorsomedial nucleus of thalamus, ventral basal ganglia, and mOFC were concordantly activated across all four modalities (i.e., visual, acoustic, tactile and osphretic) during aesthetic processing. These regions defined a general aesthetic network including the anterior insular, ventral basal ganglia, rACC and mOFC. In this article, aesthetic processing was naturalized as the appraisal of valence of perceived objects, which involves an interaction between interoceptive and exteroceptive processing. In other words, aesthetic is rooted in a comparison between subjective awareness of current homeostatic state and exteroceptive perception of objects in the environment. However, it has been argued that this is just a general cognitive process that not only can be applied to art objects but also to non-art objects[8].

Though these studies contributed significantly to exploring the general neural mechanism for aesthetics, the implications for artistic appreciation still have not been charaterized. For instance, Brown’s aesthetic model assumes an integrated neural basis for aesthetics of all sensory modalities rather than a different basis for different types, resulting in a finite identification for disparate kinds of aesthetics. Moreover, a meta-analysis research comparing the brain responses to monetary, erotic and food reward outcomes found that for the secondary reward elicited higher activation in the frontal of OFC, whereas the primary reward elicited higher activation in the frontal insular. This meta-analysis study found possible segregated regions involved in different reward processes [9]. Krik and his colleagues[10] found a similar pattern of results. Architects and non-architects were asked to make aesthetic judgments of architectural and control stimuli. The results indicated that experts and non-experts differentially recruited bilateral medial orbitofrontal cortex (OFC) and subcallosal cingulate gyrus during the task, even in the absence of a difference in the aesthetic rating made by these two groups. By contrast, activity in nucleus accumbens (NAcc) exhibited a similar response pattern. These findings suggested a dissociable role between these regions in the aesthetic evaluation.

To answer the question “why music and other artworks activate this same circuitry”, Brown et al.[8] argued that “[aesthetic processing] evolved first for the appraisal of objects of survival advantage, such as food sources, and was later co-opted in humans for the experience of artworks for the satisfaction of social needs.” Given the high correlation between regions for reward and aesthetic processing, we speculated that brain activation would also be disparate for different kinds of aesthetics. In a recent fMRI study [11], our laboratory found that facial beauty involved both the subcortical reward region putamen and the cortical reward region OFC, while moral beauty involved only the OFC. The selective activation of the ventral striatum(VS) and OFC for different types of aesthetic was suggested to represent the association between aesthetics and the physiological or social demands. Given this finding, we hypothesized that the high level artistic work that was related with social needs would elicit higher activation in the frontal cortex, whereas the low level popular work that was related with physical needs would elicit higher activation in the striatum region.

In the modernist view, genres of art develop a hierarchy[3, 12]. Artistic music, as a higher and complex form, demands an intellectual response. While popular music can not combine popularity and complexity, because popularity requires accessibility. In contrast to artistic music, popular music is more simplistic and repetitive. Therefore, popular music encourages a passive perceptual and physical engagement. It is lack of intellectual challenge and social truth. In the present study, we employed the functional magnetic resonance imaging (fMRI) to explore the neural processes of reward that arise in the appreciation of artistic and popular music. We assumed that high level music would elicit larger activity in OFC or other cortical reward region, and low level music would elicit larger activity in subcortical reward region.

## Material and Methods

### Ethics Statement

The current study was approved by the Academic Committee of the School of Psychology at South China Normal University. All participants gave written informed consent before participating in the experiments.

### Participants

Eighteen volunteers (male; 18–24 years old) were recruited from local universities for the fMRI experiment. All of them were philharmonics, but not music professional, who had engaged in some musical training or activities (e.g., choir, Musical Instruments class) before the experiment. They were right handed and reported no prior history of neurological or psychiatric problems. Participants were given a small payment after the experiment.

### Stimuli

The musical materials selected for the present research consisted of popular music excerpts, artistic music excerpts (opera), and clips of meaningless musical notes in form of playing and singing (please see supplementary material for demo, including S1 File: popular music sample, S2 File: artistic music sample and S3 File: control material sample). The former two musical materials were snipped from songs from CDs or the internet with the theme of love. These songs were all performed by female vocalists, and the lyrics were in languages other than English and Chinese. The notes clips were made in our lab and applied as control stimuli. With the GoldWave (Version 5.58, GoldWave Inc., www.goldwave.com), all stimuli were standardized to a proper length and identical volume (16dB). Each excerpt began with 500 ms of gradual fade in, and ended up with 500 ms of gradual fade out. 30 popular music, 32 artistic music and 35 notes clips were prepared for pretesting. The duration of the popular and artistic music was between 12–24 s, and that of the notes was 10–15 s. Moreover, beauty and familiarity evaluations for all these materials were collected using a 7-point Likert scale from another eighteen participants, who shared the same age and musical experience with the fMRI participants.

Based on the pretesting, 40 musical excerpts were selected by matching the beauty, familiarity and length, with half for the popular music and half for the artistic music. The familarity ratings for popular and artistic music were: 3.41±0.33 vs. 3.31±0.37; the beauty ratings for popular and artistic music were: 5.17±0.35 vs. 4.98±0.37; and the lengths for popular and artistic music were: 17.80±2.65s vs. 17.85±3.88s. For these three dimensions, there was no significant difference between popular and artistic music (*ps*>0.05,all). 28 clips of musical notes playing and singing were chosen for the baseline condition by matching the familiarity and total duration between musical (popular and artistic music) and non-musical (note) materials. The average length of notes clips was 12.14±1.27s. The familiarity ratings for note clips (3.29±0.49) were not remarkably different from those for popular and artistic music excerpts, whereas the beauty ratings of note clips (2.81±0.33) were significantly lower than those of musical materials, both *p* = .000 (LSD).

All the auditory stimuli were present binaurally with a high-quality MRI-compatible headphone system (SA-9800B, Shenzhen Sinorad Medical Electronics, Inc.). The volume of auditory stimuli was individually adjusted before fMRI scanning. Participants were asked to close their eyes during the experiment and open their eyes during the break. Visual instructions were presented on a screen back-projected on a head coil-mounted mirror.

### Procedures

The experiment program was conducted by the E-Prime 1.2 (Psychology Software Tools, Inc., Pittsburgh, PA). Prior to fMRI scanning, participants underwent a training session to become acquainted with the procedures. In the training session, the materials and the number of trials were not the same as formal experiment.

The fMRI experiment consisted of 4 consecutive scanning runs. Each run contained 17 stimuli epochs, with 5 for each musical condition and 7 for the control condition. The sequences of stimuli for the four runs were presented as follows (p = popular epoch; a = artistic epoch; c = control epoch): 1) c, p, c, a, c, a, c, p, c, a, p, p, a, c, a, p, c; 2)c, p, a, c, a, p, p, a, c, p, c, a, c, a, p, a, c; 3)p, a, c, p, c, a, c, a, p, c, a, a, c, p, c, p, a, c; 4) c, a, p, c, a, c, p, c, p, a, c, a, p, c, p, a, c. Half of the participants were assigned to the main stimuli sequence (i.e., 1,2,3,4), and half were assigned to the alternative sequence (i.e., 3,4,1,2). Each run started with a blank lasting for 4 s, and then the music excerpt was presented. During inter-stimulus interval (ISI), participants had to rate the beauty of each stimulus by pressing the corresponding key. The duration of ISI was about two thirds of the presentation time of previous stimuli. The whole experimental run lasted for 420 s without any stimuli during the last 10s. All participants were asked to close their eyes during each experimental run, and open their eyes during rest.

After the fMRI session, participants were required to rate the following two questions for all stimuli using a 7-point Likert scale (response format: 1 = "not at all"; 4 = "medium"; 7 = "extremely"): A. The beauty of the music; and B. The familiarity of the music.

### MRI Data Acquisition

MRI data were acquired using a 3T whole-body scanner (Siemens TIM TRIO). Functional images were obtained using a multislice echo plannar imaging (EPI) sequence (36 slices, slice thickness 3.5+0.7 mm gap, TR = 2.2 S, TE = 30ms, field of view = 220*220mm2, 64*64 matrix, flip angle: 90°). Scanning slices were aligned approximately parallel to the AP-PC plane, and interval scanning was carried out from the bottom up. For spatial normalization, a high-resolution T1-weighted anatomical image was acquired after EPI acquisition, using fast spin echo sequence(176 slices, 1×1×1mm, FOV = 256*256mm2, TE = 2.43ms, TR = 2530ms).

### fMRI Data Analysis

The obtained fMRI data were preprocessed and analyzed using the statistical parametric mapping (SPM8; Wellcome Trust Center for Imaging, London, UK; http://www.fil.ion.ucl.ac.uk/spm). For stabilization of magnetization, the first five volumes of each session were discarded. Data preprocessing was done with default setting of SPM8. EPI images were co-registered and normalized to the T1 standard template in Montreal Neurological Institute (MNI) space (resampling voxel size: 2×2×2mm), and smoothed with a Gaussian kernel with 6 mm FWHM.

After preprocessing, we carried out both factorial model and parametric model analyses [13]. For the factorial model, first level analysis was performed on each subject by estimating the variance of musical epoch according to a general linear model (GLM). Three kinds of musical epochs were modeled as separate regressors convolved with the canonical hemodynamic response function. For the parametric model, trials of pop music or artistic music were respectively included into a single regressor, accompanied by a parametric regressor of according post-aesthetic rating. For both models, six motion parameters estimated during the realignment procedure were included as covariates of no interest.

At the group level, all images were subjected to a voxel-wise contrast and one way ANOVA-within subject analysis to assess statistical significance for the factorial model. ROI analyses with two sample t-tests were further performed in whole brain clusters showing a significant contract between popular music and artistic music conditions. Marsbar (version 0.42) was applied to extract the beta value, with spherical ROIs of 10mm radius for putamen, 12mm radius for other areas. The central location of each ROI was determined by the results of factorial model analysis. For the parametric analysis, one-sample t-tests were used to reveal the regions in which the BOLD signal correlatively changed with the aesthetic rating scores of popular and artistic music, respectively. A tow-sample t-test was also used to find out the areas response differently to the rating of the two type of music. For the above models, global analyses were conducted at a voxel threshold of *P*<0.002 (uncorrected), and a cluster threshold of FDR<0.05. Small volume correction (SVC) was used, with a 10 mm radius centering a sphere on the coordinate of the ventral striatum peak voxel, and a 12 mm radius centering a sphere on the coordinate of the peak voxels of other regions.

The behavioral data of scanning rating, and post-scanning ratings were analyzed by the One-way ANOVA (using LSD in post hoc analysis) via software SPSS16.

## Results

### Behavioral Results

Beauty ratings during scan and post-scan were both concerned and analyzed. During fMRI scanning the beauty ratings of popular music (3.29±0.52) did not differ from those of artistic music (3.24±0.58), *P* = 0.80 (LSD); however, beauty ratings of both musical materials were significantly higher than those of control notes (1.88±0.57), both *P*<0.01 (LSD). In the post-scanning the beauty ratings for the three types of stimuli displayed the same pattern as those in the fMRI scanning, with larger ratings for popular (5.16±0.29) and artistic (5.00±0.28) music than control notes (2.21±0.55), both *P*<0.01 (LSD). The analysis of the familiarity ratings in the post-scanning was also performed, yielding no remarkable differences between these three types of stimuli, with 3.45±0.49 for popular music, 3.31±0.41 for artistic music, and 3.26±0.31 for control notes, *P* = 0.25 (one-way ANOVA).

### fMRI Results

**Fig 1:**
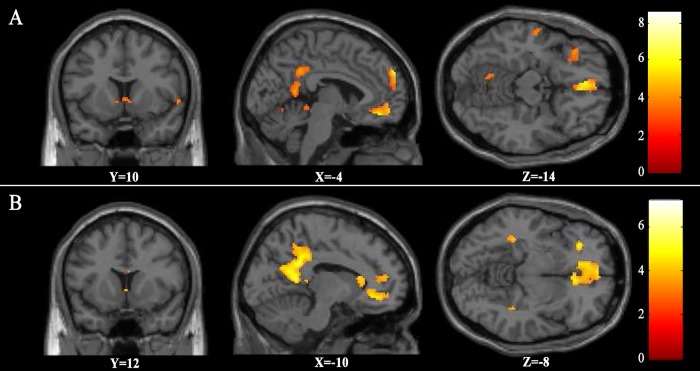
Brain activation of popular and artistic music vs. control material. Larger activity in bilateral mOFC and bilateral ventral striatum were found for popular(A) and artistic(B) music than for control material.

**Fig 2:**
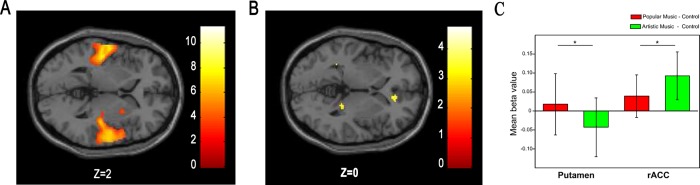
Activation of reward circuit in the contrast of popular and artistic music. (A) Popular music evoked larger BOLD response in the right putamen (26, 6,2) than artistic music; (B) Artistic music evoked larger BOLD response in the right rACC than popular music (BA24/32, 12,36,0). (C) Mean beta values and SD of the ROI analysis in putamen and right rACC for popular music and artistic music comparing control condition, respectively. Within every single ROI, beta values of both popular music and artistic music conditions were first subtracted by the mean beta value of control condition in the same ROI, before two sample t-test.

As can be seen in Fig 1, higher BOLD responses in bilateral mOFC and ventral striatum were observed for popular and artistic music than for notes. Table 1 shows coordinates, T value, and cluster sizes of the significant activation revealed by these contrasts respectively. A direct contrast between popular and artistic music demonstrated that popular music induced more activation in right putamen (see Fig 2A), and artistic music induced more activation in right rACC (see Fig 2B and Table 2). Further analyses on the beta values extracted from these two ROIs revealed that, for the right putamen, there were greater activation for popular music condition than for artistic music condition, *t*(34) = 2.30, *P* = 0.03. In contrast, for the right rACC, there were greater activation for the artistic music condition than for the popular music condition, *t*(34) = 2.68, *P* = 0.01.

**Table 1:** Regions showing a main effect at *p*<0.05 with FDR correction at the cluster level for contrasts Popular music > Notes Clip and Artistic music>Notes Clip.

Region	BA	L/R	Peak voxel	T-Value	*K*
**Popular music > Notes Clip**					
TTG	41	R	52,-22,12	8.60	1347
STG	22	R	54,-10,4	8.45	
STG	41/22	L	-46,-24,6	7.12	599
mOFC	11	L	-4,42,-14	5.85	498
	11	R	4,38,-14	5.33	
rACC	32	R	8,32,-10	4.25	
prMFC	9	L	-6,58,34	6.40	334
arMFC	10		-14,58,14	3.91	
PHG(SVC)	20	R	38,-12,-20	5.64	45
ITG(SVC)	21	L	-62,-8,-22	5.25	152
Striatum(SVC)	Caudate Head	R	2,6,0	3.88	63
	Caudate Head	L	-6,10,-2	3.53	14
**Artistic music > Notes Clip**					
SFG/MFG	8	L	-28,34,48	7.16	413
PHG/HG		R	36,-10,-20	5.84	217
HG(SVC)		L	-34,-18,-14	4.29	216
PCC	31	L	-14,-44,30	6.57	2024
	31	R	16,-42,28	5.02	
PC	31	L	-6,-66,24	4.92	
arMFC	10	L	-2,56,10	4.56	1868
mOFC	11	L	-10,42,-8	4.45	
mOFC	32	R	6,40,-10	4.24	
Striatum	Caudate Head	L/R	0,12,0	3.58	
MTG/ITG	21	L	-60,-2,-18	4.71	225
			-44,8,-28	3.27	
Cuneus	19	L	-30,-84,30	4.61	469
AG	39	L	-46,-70,34	4.49	

STG = Superior Temporal Gyrus, MTG = middle temporal gyrus, ITG = inferior temporal gyrus, TTG = transverse temporal gyri, mOFC = medial orbital frontal cortex, rACC = rostral anterior cingulate cortex, prMFC = posterior rostral medial prefrontal cortex, arMFC = anterior rostral medial prefrontal cortex, SFG = superior frontal gyrus, MFG = middle frontal gyrus, PHG = parahippocampus gyrus, HG = hippocampus gyrus, PCC = posterior cingulated cortex, PC = precuneus, AG = angular gyrus, BA = Brodmann area.

Where more than one BA is shown, the peak voxel falls in the first BA, but the cluster extends to include the others listed. L/R = left/right, peak voxel = MNI xyz co-ordinates, *k* = cluster size.

**Table 2:** Regions showing a main effect at p<0.05 with FDR correction at the cluster level for contrasts Popular music > Artistic music and Artistic music>Popular music.

Region	BA	L/R	Peak voxel	T-Value	*K*
**Popular music > Artistic music**					
TTG	41	R	52,-22,12	11.28	2404
Insula	13		40,-22,4	7.43	
STG	22		62,-12,2	6.83	
STG	41	L	-46,-34,10	8.76	2147
STG	41		-46,-24, 6	8.41	
STG	22		-58,-8,-2	8.11	
Striatum(SVC)	Putamen	R	26,6,2	3.57	20
**Artistic music > Popular music**					
PC(SVC)	7	L	-4,-42,46	4.62	239
PCC(SVC)	31	R	16,-40,36	3.97	
TPJ					
AG(SVC)	39/19	L	-32,-80,32	4.44	126
STG(SVC)	22	L	-38,-54,12	5.20	76
STG(SVC)	13	R	50,-44,18	4.19	113
HG/PHG					
PHG(SVC)	19	L	-38,-42,-8	4.32	67
HG(SVC)		L	-28,-22,-14	3.71	36
		R	30,-32,-6	3.29	54
arMFC(SVC)	32/10	L	-6,36,20	3.54	32
mPFC(SVC)					
rACC	32	R	12,36,0	3.74	25

mPFC = medial prefrontal cortex, TPJ = temporoparietal junction. Please refer to the notes of Table 1 for the rest region names in short.

BA = Brodmann area. Where more than one BA is shown, the peak voxel falls in the first BA, but the cluster extends to include the others listed. L/R = left/right, peak voxel = MNI xyz co-ordinates, *k* = cluster size.

**Fig 3:**
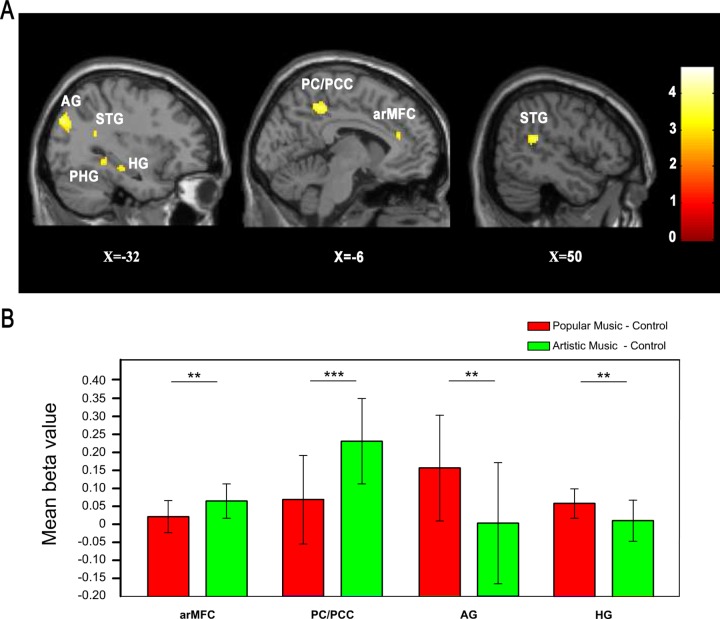
Cognitive empathy regions were activated in the contrast of artistic vs. popular music. (A) The regions consisted of left arMFC, left PC/PCC, left angular gyrus, left hippocampus /parahippocampus, and left and right inferior temporal gyrus. (B) Mean beta values and SD of the ROI analysis in left arMFC (-6,36,20), left PC/PCC (-4,-42,46), left angular gyrus (-32,-80,32) and left hippocampus (-28,-22,-14) for popular music and artistic music comparing control condition, respectively. Within every single ROI, beta values of both popular music and artistic music conditions were first subtracted by the mean beta value of control condition in the same ROI, before two sample t-test.

We also found popular and artistic music both elicited more default mode network/Theory of Mind (DMN/ToM) regions, such as arMFC, PCC/PC, temporal pole, and parahippocampal gyrus, than the notes clip (please see Table 1). Some other regions, such as angular gyrus and hippocampus, were also observed to show significantly more activation for artistic music than for control note (see Table 1). DMN/ToM regions, such as left arMFC (BA32/10), left angular gyrus, left inferior temporal gyrus, left hippocampus/parahippocampus, right hippocampus and right inferior temporal gyrus, showed greater BOLD response to artistic music than to popular music (see Fig 3 and Table 2). The result of ROI analysis also revealed the significant difference of Beta values in these areas(*P*_*arMFC*_< 0.01, *t*_*arMFC (34)*_ = 2.80; *P*_*PCC/PC*_< 0.01, *t*_*PCC/PC (34)*_ = 4.02; *P*_*HG*_< 0.01, *t*_*HG (34)*_ = 2.89; *P*_*AG*_< 0.01, *t*_*AG (34)*_ = 2.91).

**Fig 4:**
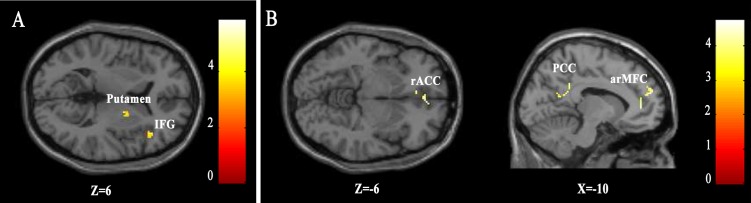
Cerebral regions tracking increasing aesthetic rating of popular music and artistic music. (A) Cerebral regions tracking increasing rating of popular music included right putamen and IFG. (B) Regions tracking increasing rating of artistic music included right rACC, left arMFC and PCC.

The parametric analysis revealed similar results. For popular music, as the post aesthetic ratings increased the activation level of right putamen increased (see Fig 4A and Table 3). The activity of right interior frontal gyrus and some cerebellar area such as culmen, tonsil and declive also showed linear relationships to aesthetic ratings. We also found a cluster located in supplementary motor area (SMA) in which the activity tracked with aesthetic ratings of popular music. However, the small volume correction only revealed a marginal significant(*P*_*FWE-corr*_ = 0.058). For artistic music, we found positive linear correlation between the aesthetic ratings and the activation level in several regions, including rACC, arMFC and PCC(see Fig 4B and Table 3). However, we can not found any brain regions tracking aesthetic ratings of popular music more synchronously than those of artistic music or regions tracking aesthetic ratings of artistic music more synchronously than those of popular music.

**Table 3:** Regions tracking increasing aesthetic ratings of popular music and artistic music.

Region	BA	L/R	Peak voxel	T-Value	*K*
**Popular music**					
Putamen(SVC)		R	22,-2,0	5.04	46
IFG(SVC)	45/13	R	44,26,8	4.25	52
Cerebellum					
Culmen/ Tonsil(SVC)		L	-10, -42, -26	5.91	139
Tonsil(SVC)		R	14–56–40	4.82	75
Declive(SVC)		L	-10–72–24	4.52	250
**Artistic music**					
arMFC(SVC)	BA9/10/32	L	-16 48 14	4.55	106
rACC(SVC)	BA10/32	R	6 48–6	4.42	44
PCC(SVC)	BA31	L	-12–44 30	4.12	33

IFG = Inferior Frontal Gyrus. Please refer to the notes of Table 1 for the rest region names in short.

BA = Brodmann area. Where more than one BA is shown, the peak voxel falls in the first BA, but the cluster extends to include the others listed. L/R = left/right, peak voxel = MNI xyz co-ordinates, *k* = cluster size.

## Discussion

In the present study, artistic music, popular music and musical notes playing and singing were manipulated to investigate the general and specific neural correlate for acoustical aesthetic. The artistic music was related with higher VS activation compared with control notes. Moreover, the artistic music activated more right mPFC than popular music. These findings indicated the mechanism differences between artistic music and non-artistic music reflects in the reward circuits. In the previous studies focused on neuroaesthetics, parameter or category design was commonly applied, and they focused on the form beauty based on abstract graphics [14], external beauty based on facial stimuli [15–18], and art beauty based on painting, music, dancing, and sculpture stimuli [5, 6, 19–21]. Two reward circuits were found to be involved in processing of different kinds of beauty: one is the cortical reward circuit (includes mOFC); another is the sub-cortical reward circuit, such as striatum (includes putamen, caudate, and nucleus accumbens). However, inconsistent activation patterns of these two circuits for aesthetics were reported in previous research. For facial beauty, nucleus accumbens and OFC were separately found to be activated in the condition of facial beauty in some research[15, 17, 18]; whereas in some other studies, these two regions were simultaneously activated for beautiful faces [16, 22, 23]. For painting beauty, Vartanian and Goel found that the BOLD response of caudate was decreased as the likable ratings got smaller [24]; Kavabata and Zeke [5] reported a larger activation of OFC during the appreciation of a beautiful painting. Moreover, OFC and caudate were simultaneously activated when viewing beautiful paintings in other research [6, 7].

These findings demonstrate possible diverse activation patterns in cortical and sub-cortical reward circuits for different aesthetics, which raises a further question about which factors would determine the connection between aesthetics and brain activity. Most recently, an attempt by Wang and Mo [11] produced some exciting findings and should shed light on this issue. They compared the networks of moral beauty and facial beauty, and found that moral beauty representing advanced social needs recruited only the cortical reward region OFC, whereas facial beauty recruited both the OFC and the subcortical reward region putamen. Given that, we supposed that the correlation between aesthetic objects and the physical (basic) or social (advanced) demand would determine the activation pattern of prefrontal reward region and striatum in reward circuit. That is, the more the aesthetic object relied on basic demand, the more VS would be involved; the more the aesthetic object relied on advanced social demand, the more prefrontal reward regions would be involved.

The present research was conducted to further verify our aesthetic-demand hypothesis by comparing the appreciation process between artistic music (high level) and popular music (low level) via fMRI technology. As expected, popular and artistic music both elicited larger activation in VS than control note, and popular music evoked larger activation in right putamen than artistic music. The parametric analyses also revealed that the activation of putamen tracked aesthetic ratings of popular music significantly more synchronously than those of artistic music. For the cortical circuit, popular and artistic music both elicited larger BOLD response in mOFC (BA11) than control note. Moreover, artistic music evoked larger BOLD response in right rACC (BA32) than popular music. The BOLD signal in right rACC and the adjacent anterior prefrontal cortex (aPFC, BA10) also showed higher correlation with the aesthetic rating of artistic music than with the popular music.

mOFC and rACC were found activated in response to the reward stimuli[6, 25–27]. In a study about cross-modality aesthetic, the activity of mOFC in BA11 was observed during visual or acoustical art appreciation. The nearby regions such as rACC (BA32) and aPFC (BA10) also showed sensitivity to the beauty[6]. The latter two regions were collectively considered as medial prefrontal cortex (mPFC) [27, 28], which would also be preferentially recruited by the value of rewards as mOFC [25–27]. It is worth noting that, in the present study, the beauty ratings for artistic and popular music were not significantly different. Therefore, the beauty rating difference can not be responsible for the higher activation of mPFC for artistic music than for popular music. Considering the parametric analysis results which suggested that the activation level of mPFC was much more correlative with artistic music rating, we propose that the increased mPFC activity for artistic music would derive from the greater affection for artistic stimuli. Our view agreed with the results of Trost and colleagues in which they examined the neural correlates for disparate musical emotions[29]. In this study, emotions, such as tenderness, peacefulness, transcendence, and nostalgia, which were characterized by sublime aesthetic also features recruited more rACC than other emotions. Alternatively, rACC has been considered to serve a role in recruiting greater attentional control for emotion processing[30, 31]. Under these circumstances, the activation of rACC in artistic music appreciation in present study might reflect more intensive intellectual efforts. In summary, the results of present study supported our first hypothesis that the high level artistic work, which was relevant to social needs or more intensive intellectual response, would elicit higher activation in the frontal cortex, whereas the low level popular work, which was relevant to physical needs, would elicit higher activation in the striatum region.

In addition, we also found that artistic music evoked higher arousal in the default network/ToM than popular music, which indicated larger involvement of social cognition for artistic music appreciation. Empathic engagement between an audience and art works is considered as another crucial element for artistic appreciation[32–34], especially in the music domain. From this point of view, an audience could understand or experience the intention, affective and feeling implicated in the artworks through empathy. Based on the studies of normal and brain lesion participants, two types of empathy—emotional empathy and cognitive empathy—have been identified[35, 36]. Emotional empathy is a pure emotional contagion, which relies on the mirror neuron system (MNS), while cognitive empathy is more advanced and relies on the mentalizing or theory of mind (ToM) system. Although default network/ToM regions were also found to be activated during art work appreciation in previous studies, activation in these areas was never considered to reflect cognitive empathy [7, 29, 37]. For example, in a study of Brown and colleagues[37], participants were asked to listen to beautiful but unfamiliar music, which elicited activation in the left rACC (BA32), retrosplenial cortex (BA29/30), and hippocampus. The activation of these regions was interpreted as the representation of emotion processing. In another work of musical appreciation, sublime music evoked the activity of vmPFC and hippocampus/ parahippocampus, this finding was determined to be an automatically associative processes for this kind of music [29]. But more and more evidence verifies the possible association between the default mode network for art appreciation and social cognition. In the study of Geday and Gjedde [38], strong emotion caused a decrease of the deactivation of the arMFC during the task. Moreover, this effect disappeared in the situation without self-involvement, which demonstrated the possible influence of social cognition on arMFC activation. More recently, Vessel [7] found that the most moving art works were related to the higher arousal in arMFC, PCC, and hippocampus. These findings were attributed to the personal relevance during aesthetic experience. Aesthetic was proposed as a process that requires personal relevance to combine the perception and emotional reaction. Given that, the larger activation for artistic music than for popular music in default network/ToM related regions should not represent the beauty difference between these two types of music, because no significant difference in beauty rating were found in our study. The parametric analysis results in this paper also gave additional evidence for the role of these areas in music appreciation. These results also exclude the possibility that the DNM have just been recruited in artistic music condition for a simple music monitoring task, even given that DMN is also relevant to internal goal or simple tasks. In summary, the more sensitive response in DMN/ToM related regions to artistic music appreciation than popular music appreciation would represent the greater involvement of advanced social cognitive empathy for artistic music than popular music.

## Conclusions

This study applied fMRI technology to explore the disparate neural activations in appreciation of popular and artistic music. Both sub-cortical (e.g., VS) and cortical (e.g., vmPFC) reward regions engaged in artistic and popular music aesthetic appreciation, while the sub-cortical reward region (e.g., putamen) was more sensitive to popular music while the cortical region (e.g., mPFC) was more sensitive to artistic music. In addition, the cognitive empathy regions, including PCC/PC, TPJp and arMFC, were more responsive to artistic music than popular music and control notes, implying more social cognition involved artistic music aesthetic appreciation. In conclusion, this study gives clear neuronal evidences supporting the view that artistic music is of intelligence while the popular music is of physiology.

## Supporting Information

S1 File Popular music sample. (WAV)

S2 File Artistic music sample. (WAV)

S3 File Control material sample. (WAV)
